# Supplementary material for: How patients experience nurse-doctor collaborative care at specialist clinics: A qualitative study
Source: PLoS One. 2025 May 9;20(5):e0321192. doi: 10.1371/journal.pone.0321192 (PMC12064018; doi:10.1371/journal.pone.0321192)
Supplement: S1 Appendix — (DOCX) [file pone.0321192.s001.docx]

Name of observer: Date of observation: Location of observation Page:

| Time stamp | HCP interacting with patient | Observe: how patient-centred care is delivered. | Reflections by the observer of patient-centred care:   1. Patient is treated as a person (versus a mere carrier of a disease) 2. Holistic care versus only biomedical perspective) 3. Therapeutic alliance between HCP and the patient |
| --- | --- | --- | --- |
|  |  |  |  |
|  |  |  |  |
|  |  |  |  |
|  |  |  |  |
