# Supplementary material for: How patients experience nurse-doctor collaborative care at specialist clinics: A qualitative study
Source: PLoS One. 2025 May 9;20(5):e0321192. doi: 10.1371/journal.pone.0321192 (PMC12064018; doi:10.1371/journal.pone.0321192)
Supplement: S2 Appendix — (DOCX) [file pone.0321192.s002.docx]

Preliminaries:

(Hi Mr or Ms xxx. Thanks for speaking to us today. I’m Xiaohui, and I met you when you went to see Dr so-and-so on 8 Feb. Qianhui and Yang Yann to intro themselves.)

Thank you for giving us your time. We want to interview you to understand how you have experienced the care for your (condition). This is to help us improve the care for other patients in future.

May we also get your permission to record this interview? It’s to help us accurately transcribe what you say. Thank you very much.

1. Could you share with us why you went to see Dr (so-and-so)?

2. How are you doing now?

3. When we met on (date), you saw the nurse first, then you saw Dr (so-and-so). How did you feel about seeing the nurse first?

4. What did the nurse talk to you about?

5. How did you feel about receiving care advice from the nurse?

6. What did the doctor talk to you about?

7. How did you feel about receiving care advice from the doctor?

8. What do you think is the role of a nurse in the care of your (condition)?

9. What do you think is the role of the doctor in the care of your (condition)?

10. How do you feel about being seen by both the nurse and doctor?

11. Who do you think is more important to your care and why?

12. How do you feel about the care you’ve received? Could you give us some examples?

13. Is there anything you’d like to share with us that we’ve not discussed so far?
