# Supplementary material for: How patients experience nurse-doctor collaborative care at specialist clinics: A qualitative study
Source: PLoS One. 2025 May 9;20(5):e0321192. doi: 10.1371/journal.pone.0321192 (PMC12064018; doi:10.1371/journal.pone.0321192)
Supplement: Attachment — Submitted filename: Response to Reviewers.docx [file pone.0321192.s006.docx]

| **Reviewer’s comments** | **Authors’ response** |
| --- | --- |
| **Reviewer 1** | |
| 1. Please provide any specific reference on how you decided that a sample of 27 with a response of 12 is enough for generating valid results of your qualitative research work. | The specific Braun and Clarke reference [1] we have used is found in lines 183-193 of the original manuscript. In the revised manuscript with tracked changes, this is found in lines 214-225. Thank you. |
| 2. How was an agreement reached to establish consensus on the themes among the authors? | Thank you for highlighting our manuscript’s inadequacy in explaining the process by which we constructed, revised and finalized the themes in the study, especially when interpretive variations arose.  The changes are found in lines 240-247 of the revised manuscript with tracked changes. |
| **Reviewer 2** | |
| The abstract effectively outlines the study’s major themes, highlights its focus on thematic analysis, and summarizes the main findings, all while adhering to word count requirements. However, refinement in phrasing is recommended. For instance, while the abstract identifies major points of the study, the statement “major themes within article and guidelines for thematic analysis” could be more explicitly tied to specific findings to improve clarity and impact. | Thank you for your compliment and suggestion.  The changes are found in lines 43-58 of the revised manuscript with tracked changes. |
| Critique of the Introduction include:  Ambiguity in Sentence Structure: The sentence, “While some patients appreciated the holistic support offered by the nurse-doctor collaborative care model, patients whose disease status was still active seemed eager to control their symptoms through drug treatment managed by the doctor,” lacks clarity. It implies a dichotomy in patient experiences but does not clearly connect these sentiments to broader themes or findings. Contextualizing in discussion or rephrasing is recommended to ensure the point is well-articulated and supports the study’s framework. | The changes are found in lines 43-58 of the revised manuscript with tracked changes. Thank you. |
| Conclusion Statement: The concluding assertion, “Interprofessional collaboration enhances patient care in specialist clinics,” reads more as an opinion rather than a conclusion supported by evidence from the analysis. Consider revising this statement to objectively reflect specific findings or themes emerging from the study.  Specific Recommendations:  -Clarify the sentence about patient perspectives to ensure it aligns with the study's analytical framework and contributes to the broader discussion of PX in IPC care. | The changes are found in lines 60-61 of the revised manuscript with tracked changes. Thank you. |
| 2. Novelty of Results  While there are opportunities to reference the study as a more novel contribution to literature, the presented work builds upon existing research on interprofessional collaboration in primary care settings. However, the study's focus on specialist contexts is less extensively explored in the literature. This suggests a novel contribution to the field by extending current understanding to a relatively understudied area  Specific recommendations:  More explicitly state the novelty of the research in areas of application for reader to understand or in its role of solely being explored in specialty settings. | The changes are found in lines 113-117 of the revised manuscript with tracked changes. Thank you. |
| 3. Technical Standards and Methodology  Specific recommendations for methodology that require further attention:  The inclusion of specialist clinics engaging in interprofessional collaboration (IPC) practices is valuable, however, recommend a more detailed description/rationale of the criteria used for specialty clinic selection. | The rationale for studying these three specialty clinics was explained in lines 109-111 and 117-119 in the original manuscript (lines 122-123 and 129-131 in the revised manuscript with tracked changes).  In summary, this study was an extension of our previous studies (references #17 and #18) which aimed to understand the facilitators and barriers of implementing IPC at a specialist care center. We kept the study setting to the same three clinics as our knowledge gained from the previous study provides useful context to inform our interpretation of the patient experience in these clinics. Thank you. |
| The statement regarding SingHealth Central Institutional Review Board exemption should be clarified to include what specific data categorization applies to this. | The changes are found in lines 141-144 of the revised manuscript with tracked changes. Thank you. |
| The definition of "inactive" in the context of disease state should be further defined for period disease inactivity (e.g., no flare-ups within the past 12 months). | The changes are found in lines 160-161 of the revised manuscript with tracked changes. Thank you. |
| The study should provide more specific details on how the InterPACT framework was applied to categorize IPC within the context of the study's focus and its usage by healthcare professionals (HCPs) in self-assessment. | The changes are found in lines 186-190 of the revised manuscript with tracked changes. Thank you. |
| The statement regarding the researchers' efforts to be unobtrusive in patient interactions should be further elaborated upon to clarify the extent of their observation during patient encounters. | The changes are found in lines 202-204 of the revised manuscript with tracked changes. Thank you. |
| Consistent use of grammar tense: Recommend consistent use of past tense throughout. The statement regarding the overlap between the first two themes requires rephrasing for improved clarity. In acknowledging the potential influence of researcher positionality on data interpretation, the use of "may have" instead of definitive statements would be more appropriate (e.g., "Non-HCP members' interpretations of the data may have been based on their positionalities…")." | We have adopted the reviewer’s recommendation to use past tense where appropriate throughout the revised manuscript with tracked changes.  However, we have retained the use of the present perfect tense in certain instances in the Introduction and Discussion sections. We felt this would be appropriate when referring to actions or events that began in the past but which still continue to have relevance at the present time.  We would prefer to keep the past tense for the sentence, “Non-HCP members’ interpretations of the data were based on their positionalities as health services…researchers”, as it was an action we had completed in the past. Thank you. |
| 4. Conclusions and Data Support  The following are specific recommendations:  -Study “strengths” section: Currently there is not a section referencing study strengths. The article could be strengthened by a more explicit discussion of study strengths. The acknowledgment of reflexivity in the research process is a notable strength. | Our paper has explicitly stated how our study adds to the literature (lines 439-532 of the revised manuscript with tracked changes). As such, we will not be adding a section on strengths. Thank you. |
| -The choice of methodology, including the maximum variation selection strategy, requires a more explicit discussion of its strengths and limitations. | The changes are found in lines 154-156 of the revised manuscript with tracked changes.  As we subscribe to the contextualist ontology [1], we do not think of reflexivity as a form of bias that is a methodological limitation. Instead, we use reflexivity to make explicit how our backgrounds, experience and knowledge inform our researcher subjectivity in the process of analyzing qualitative research data [1]. Thank you. |
| -Recommend the article acknowledge the potential limitations of reflexivity in the research process. | As we subscribe to the contextualist ontology [1], we do not think of reflexivity as a form of bias that is a methodological limitation. Instead, we use reflexivity to make explicit how our backgrounds, experience and knowledge inform our researcher subjectivity in the process of analyzing qualitative research data [1]. Thank you. |
| -Recommend the use of the term "research story" to describe data analysis be replaced with more scientifically rigorous terminology. | The changes are found in line 223 and 283 of the revised manuscript with tracked changes. Thank you. |
| There is not a clear definition of "Patient Experience" and the criteria used to differentiate between "positive" and "negative" patient experiences. | Our definition of “Patient Experience” was drawn from the paper by Ahmed and colleagues [2]. We quoted their definition in lines 150-152 of our original manuscript (lines 170-172 of the revised manuscript with tracked changes).  We included the criteria used to differentiate between “positive” and “negative” patient experiences in lines 176-179 of the revised manuscript with tracked changes. Thank you. |
| -Caution and recommendation to restate use of descriptive terminology, such as "eager" or "reticent," to infer patient motivations to be carefully considered and contextualized to avoid potential misinterpretations. | The changes are found in lines 45-50, 342-345, 369 and 434 of the revised manuscript with tracked changes. Thank you. |
| -Lines 408 - 414 - Re-state terms like "the patient," "these patients," and "this group of patients," making it difficult to understand which specific demographic is being referred to. More consistent and precise descriptions of patient demographics are crucial to avoid misinterpretations. | The changes are found in lines 450-457 of the revised manuscript with tracked changes. Thank you. |
| -Line 422 - The use of the term "older patients" without defining the age threshold should be avoided. | The changes are found in lines 465, 495 and 497-498 of the revised manuscript with tracked changes. Thank you. |
| -Line 431 - The statement regarding potential reticence among patients of Asian ethnicity should be further contextualized with specific references to relevant cultural norms within the medical community, if this is applicable to meaning. | The changes are found in lines 478-479 of the revised manuscript with tracked changes. Thank you. |
| -Line 443 - the phrase "Getting patients ready..." should be rephrased or contextualized further, as the definition of "patient preparedness" is not clearly defined in the article. | The changes are in lines 490-491 of the revised manuscript with tracked changes. Thank you. |
| Further clarification is needed regarding the statement: “SingHealth Central Institutional Review Board provided review exemption for this study (CIRB Ref: 2023/2548).” Specifically, the authors should clarify whether this exemption impacted participant consent or any other ethical considerations related to the study. | In lines 129-131 of the original manuscript, we stated that consent was taken. This is found in lines 145-148 of the revised manuscript with tracked changes. Thank you. |
| **Reviewer 3** | |
| Start line 65: I don’t see the necessary to abbreviate the “patient experience” into PX. | We have replaced PX with patient experience throughout the revised manuscript (with tracked changes and clean copy). Thank you. |
| Lines 73-74: “However, definitive association between IPC and positive patient outcomes have not been demonstrated in systematic reviews”: I think this sentence is only proper when this current study then conducted a systematic review rather than a qualitative study. Consider to paraphrase or remove “…in systematic review”. | The changes are found in line 85 of the revised manuscript with tracked changes. Thank you. |
| Please move Table 1 to be cited somewhere in the results (analysis) section instead in method. | For the purpose of keeping the flow in the manuscript, we would like to keep Table 1 in the Methods section. Thank you. |
| I did not see the reporting guidelines of this paper. Consider to look at Equator Network or COREQ (mentioned in the Equator as well). In COREQ, for example, the authors need to explain the credentials of the researchers and their experience/training in qualitative research methods. It is including who conducted the interviews and data analysis (not only their initials, but the background and their experience in conducting a qualitative studies). Even COREQ mention about the gender of the interviewer and data analysts. | We mentioned using the reflexive thematic analysis reporting (RTARG) guidelines in lines 225-226 in the original manuscript (lines 264-265 of the revised manuscript with tracked changes):  “Our analysis adhered to Braun and Clarke’s thematic analysis reporting guidelines [36] (S3 Appendix) congruent with our research values [33]”. |
| Line 172: “….averaging 5500 words” please consider to move some parts of the method section into the analysis (as commonly known as results). I found some duplication (such as the mention of Table 1” both in the method and analysis. Therefore, the authors need to consider which parts are methods, and which parts more suitable for the results section. | Our placement of line 172 was based on RTARG.  Table 1 was mentioned again in the Results section to facilitate readers’ recall of the participants’ details. Thank you. |
| Line 179: “…transcribed verbatim by QC” is there any supporting device for the transcribing process? | The transcriptions was performed manually by QC. Please see line 211 of the revised manuscript. Thank you. |
| Lines 190-192: Also consider to move this part to analysis (results). | We would like to follow RTARG and keep lines 190-192 in the Methods section. Thank you. |
| How about the trustworthiness of the data? It should be described somewhere in the method section. | Thank you for pointing this out. Trustworthiness is a very important quality criterion for qualitative research. The changes made are found in lines 267-268 of the revised manuscript with tracked changes. |
| Start from line 272: Please delete all of the name of the participants for the confidentiality, and consider to use a particular code, i.e. P1 for Participant 1. | The names used are all pseudonyms which protect the confidentiality of the participants (Table 1 column 4 line 1; line 277 of revised manuscript with tracked changes). Thank you. |

References

1.	Terry G, Hayfield N, Clarke V, Braun V. Thematic Analysis. In: Willig C, Stainton Rogers W, editors. The SAGE handbook of
 qualitative research in psychology. London: Sage; 2017. p. 17-37.

2.	Ahmed F, Burt J, Roland M. Measuring patient experience: concepts and methods. Patient. 2014;7(3):235-41.
